# How communication affects prescription decisions in consultations for acute illness in children: a systematic review and meta-ethnography

**DOI:** 10.1186/1471-2296-15-63

**Published:** 2014-04-08

**Authors:** Christie Cabral, Jeremy Horwood, Alastair D Hay, Patricia J Lucas

**Affiliations:** 1 Centre for Academic Primary Care, School of Social and Community Medicine, University of Bristol, 39 Whatley Road, Bristol BS8 2PS, UK; 2 School of Policy Studies, University of Bristol, 8 Priory Road, Bristol BS8 1TZ, UK

**Keywords:** Communication, Respiratory tract infection, Child health, Primary care, Parent, Antibiotics

## Abstract

**Background:**

Communication within primary care consultations for children with acute illness can be problematic for parents and clinicians, with potential misunderstandings contributing to over–prescription of antibiotics. This review aimed to synthesise the evidence in relation to communication and decision making in consultations for children with common acute illness.

**Methods:**

A systematic search of MEDLINE, EMBASE, CINAHL, PsycINFO, SSCI, SIGLE, Dissertation Express and NHS economic evaluation databases was conducted. Studies of primary care settings in high income countries which made direct observations of consultations and reported qualitative data were included. Included studies were appraised using the process recommended by the Cochrane Qualitative Methods Group. Credibility was assessed as high for most studies but transferability was usually assessed low or unclear. Data were synthesised using a meta–ethnographic approach.

**Results:**

Thirty–five papers and 2 theses reporting on 13 studies were included, 7 of these focussed on children with respiratory tract infections (RTI) and the remaining 6 included children with any presenting illness. Parent communication focussed on their concerns and information needs, whereas clinician communication focussed on diagnosis and treatment decisions. During information exchanges, parents often sought to justify the need for the consultation, while clinicians frequently used problem minimising language, resulting in parents and clinicians sometimes talking at cross–purposes. In the context of RTIs, a range of parent communication behaviours were interpreted by clinicians as indicating an expectation for antibiotics; however, most were ambiguous and could also be interpreted as raising concerns or requests for further information. The perceived expectation for antibiotics often changed clinician decision making into clinician–parent negotiation.

**Conclusions:**

Misunderstandings occurred due to parents and clinicians talking at cross purposes about the ‘seriousness’ of the illness and because parents’ expressions of concern or requests for additional information were sometimes perceived as a challenge to the clinicians’ diagnosis or treatment decision. This modifiable problem may be an important contribution to the unnecessary and unwanted prescribing of antibiotics. Primary care clinicians should be offered training to understand parent communication primarily as expressions of concern or attempts at understanding and always to check rather than infer parental expectations.

## Background

Acute illnesses are frequent in children and the commonest reason for use of primary health care services by parents in the UK [1] and internationally. Acute cough with respiratory tract infection (RTI) is the most common of these and is estimated to be the cause of over a million UK consultations per year [2]. Despite this they are often perceived by parents as problematic consultations [3,4]. Parents have been reported to leave consultations feeling uncertain, with insufficient information about diagnosis and treatment options [4,5]. Clinicians want to provide the best health care for their patient, whilst also satisfying parents [6]. Since acute illnesses are usually self–limiting, from a health service point of view, many of these consultations may be regarded as unnecessary, and contribute to over–prescription of antibiotics [7]. Primary care clinicians are responsible for 80% of all antibiotic prescriptions [8] and they continue to be widely prescribed despite evidence of limited effectiveness [9], contributing to the increasing rates of bacterial resistance to antibiotics [10]. The content of these interactions is therefore of importance for parents, children, clinicians and health services.

Paediatric consultations are complicated by the triadic nature of the doctor patient interaction: the needs of the patient are usually interpreted for the clinician by a third party (the parent) [11,12]. Parents commonly speak for their child and the perceived needs and anxieties of the parent can inadvertently be given priority over the child’s [13]. Parents’ communication behaviours may lead clinicians to overestimate parents’ expectation for antibiotics [14,15]. In addition, clinician communication behaviours, such as negative or positive framing of treatment recommendations can influence whether or not parents accept or resist, which in turn may influence the decision to prescribe antibiotics [16]. Training in communication skills has been used to address the problem of over–prescription of antibiotics, with some success in consultations for adults [17-19] and encouraging results in consultations for children [20].

Other reviews have looked at clinicians’ views and experiences of prescribing [21], antibiotic prescribing for the general population [6,22], triadic communication in paediatric consultations [23] and the effectiveness of interventions to influence antibiotic use for acute RTI in children [24], but none have looked at the interaction within consultation for acute childhood illness and how it affects decision making. This review will examine what happens within the consultation from the perspectives of both clinician and parent. It will look at parent–clinician communication and the processes involved in decision making (particularly in relation to prescribing or not prescribing antibiotics).

This review is part of a NIHR funded Programme Grant for Applied Research, designed to better understand and respond to childhood RTIs presenting in primary care, known as the “TARGET” Programme.

## Methods

Standard methods for systematic reviews were used to search, screen, and review included papers [25]. A study protocol was written for this review and is available from the study authors.

### Literature search & study selection

The databases MEDLINE, EMBASE, CINAHL, PsycINFO, SSCI, SIGLE, Dissertation Express databases and the NHS economic evaluation database were searched using a form of the following strategy: [terms for acute childhood illness] AND [terms for parent or child] AND [terms for clinicians or primary health care] AND [terms for consultation] AND [terms for qualitative research]. Terms for acute illness were limited to human child when possible. Search strategies were tailored to each database; the Medline search strategy is given in Additional file 1 (SM1) and other strategies are available from the authors. No date limits were set and all database records up to October 2012 were searched.

The following journals were selected for hand searching because it was anticipated that these journals would publish potentially relevant qualitative studies: Social Science and Medicine, the Sociology of Health and Illness, British Journal of General Practice, Journal of Family Practice, and Health Expectations. We used reference lists of relevant studies, citation tracking, and contacts with experts in the field to identify additional studies.

Title and abstracts (where available) were screened against study inclusion criteria and full texts of any potentially relevant studies were retrieved. Full text screening was carried out independently by 2 reviewers (CC and PL/JH) to determine inclusion in the systematic review and any disagreements were resolved by discussion. Studies were included if they 1) concerned the interaction between parent and health professional during a primary care consultation for an acute minor illness in a child; 2) took place in primary health care settings in OECD high–income countries; 3) made direct observations of the consultations; and 4) reported qualitative data. Studies reporting only quantitative data were excluded since this review sought to examine the actual words exchanged in these consultations.

### Data extraction

Data extraction was carried out independently by two reviewers (CC and PL/JH) according to the agreed protocol. Two reviewers extracted data from each study into a pre–agreed form which covered the study aim, sampling strategy, methodology, setting, population characteristics, first and second order constructs and conclusions. The two forms completed for each study were then merged and any disagreements were discussed and resolved by consensus.

### Critical appraisal

The quality assessment follows the approach recommended by the Cochrane Qualitative Research Methods Group [26]. The inclusion/exclusion criteria were used to identify studies for inclusion. Technical and theoretical aspects of the included studies were appraised independently by 2 members of the research team (CC and PL or JH) using the modified quality measure proposed by Popay et al. [27]. Quality criteria were not used to exclude studies from the review as no papers were considered “fatally flawed” [28,29].

### Data synthesis

We attempted to produce a new understanding of the data drawn from the primary studies using a meta–ethnographic approach as developed by Noblit and Hare [30] and following the iterative process described by Malpass et al. [29]. We began by identifying the second order constructs (themes or phenomena identified by the authors of each included study). This was completed independently by two authors (CC and PL or JH) as part of data extraction and a final list of second order constructs for each study was arrived at through discussion and consensus. These second order constructs were then translated across all the studies [31]. Taking each paper in chronological order, the second order constructs were entered into a table in the original authors’ words or a close paraphrase. Where similar constructs were identified in different studies a translation or summary description was written which captured the common meaning and preserved the study authors’ conceptual interpretations [29]. The ‘line of argument’ approach described by Noblit & Hare [30] was used to synthesise the translated constructs, or where these only occurred in one study the extracted second order constructs. This approach allows us to construct an argument about what these studies say together, although different studies or groups of studies were focussed on different phenomena [32].

## Results

**Figure 1:**
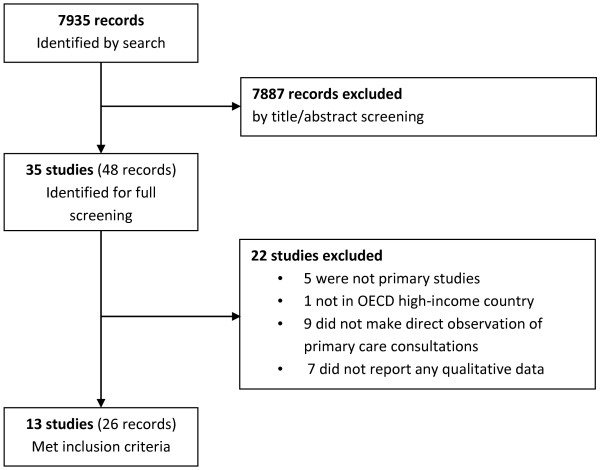
Study identification flow chart.

The search identified 7,935 unique records, of which 7,887 were excluded on screening of title and abstract. The remaining 48 records reported on 35 studies, for which full texts were obtained and screened. This identified 13 studies which met the inclusion criteria (see Figure 1). These 13 studies were reported across 35 publications and 2 theses (where studies were reported across multiple publications, we use the earliest publication as the primary reference for clarity, but in results we refer to the publication from which data were drawn where appropriate). All publications associated with each study are listed in Table 1.

**Table 1:** Study characteristics

					**Consultation sample**					
**Primary Reference**	**Associated publications**	**Focus**	**Data collection methods**	**Analysis methods**	**No.**	**Paediatric or mixed**	**Illness**	**Location**	**Consultation participants**	**Study quality**
Elwyn 1999 [33]	[34]	Shared decision making in situations of conflict	Two case studies. Audio recorded consultations.	Discourse analysis	2	Paediatric	URTI	UK	2 Children (2–8 yrs) 3 Parents 1 GP from 1 clinic	High credibility. Insufficient information to assess typicality. Transferability limited to similar cases.
Stivers 2000 [35]	[12,14,15,36-40]	Communication practices used by parents and paediatricians	Convenience sample. 295 audio recorded & 65 video recorded consultations. 1996–1997.	Conversation analysis	360	Paediatric	RTI	USA	Children (2–10 yrs) Parents (Demographic data for 295: avg. age: 38 yrs; avg. edu: 16 yrs; 75% affluent households; 69% White; all English speakers) 14 Clinicians from 6 clinics	High credibility. Likely to be typical. Limited transferability to similar populations.
Rollnick 2001 [41]		Language, skills and strategies used in everyday URTI consultations	Audio recorded consultations.	Verbal ‘moves’ used by doctors identified.	29	Paediatric	URTI	UK	Children (<11 yrs) Parents 5 GPs from 1 clinic	Insufficient information to assess credibility, transferability and dependability.
Main 2001 [42]	[43,44]	Effects of family context on care and physician-patient communication	Purposive selection of clinics. Direct observation of consultations. 1996 & 1999	Emerging patterns of physician-patient interaction were identified.	37	Mixed	Acute RTI	USA	Children (<16 yrs) Parents >50 Clinicians from >18 clinics	High credibility. Insufficient information to assess transferability of these findings.
Barry 2001 [45]	[46,47]	Patient expectations, consultation behaviour and prescription	Purposive sample of clinicians. Audio recorded consultations & interviews with parents and clinicians. 1996–1998.	Conversation analysis	35	Mix	Mix	UK	6 Children (<12 yrs) Parents 20 GPs from 20 clinics	High credibility. Insufficient information to assess typicality and transferability.
Tates 2002 [48]	[49,50]	Co-construction of roles and interaction	Video recorded. 3 time periods: 1975–78; 1988–89; 1993	Conversation analysis	106	Paediatric	Mix	Netherlands	106 Children (<12 yrs) 106 Parents (88 mothers) 58 Clinicians	High credibility. Insufficient information to assess transferability.
Butler 2004 [51]		GPs’ current practice regarding prognosis	Convenience sample. Audio recorded consultations.	Prognosis communication extracted.	59	Paediatric	RTI	UK	Children (<11 yrs) Parents 9 GPs from 2 clinics	Insufficient information to assess credibility, typicality or transferability.
Roberts 2005 [52]		Method of theme oriented discourse analysis	Two case studies. Audio or video recorded consultations.	Discourse analysis	2	Mix	Mix	Not stated	1 Child 1 Parent 1 Clinician	High credibility. Insufficient information to assess transferability or typicality.
Nova 2005 [53]		Quality of the paediatric interaction.	Videos recorded consultations. 2003.	Discourse analysis	10	Paediatric	Mix	Italy	10 Children (2–6 yrs) >6 Parents Clinicians (no information given)	High credibility. Insufficient information to assess transferability or typicality.
Stivers 2005 [16]	[54-59]	Parent resistance to no antibiotic treatment	Cross-sectional sample. Video recorded consultations. 2000 & 2001.	Conversation analysis	309	Paediatric	URTI	USA	Children (6 m to 10 yrs) 543 Parents (avg. age: 34 yrs; 53% latino; 28% white; 12% African-American; 7% Asian; 16% high school not completed; 60% high school completed; 24% graduates) 38 Paediatricians from 27 clinics	High credibility. Insufficient information to assess transferability or typicality.
Cahill 2007 [11]		Child participation in consultations	Purposive sample of practices. Video recorded consultations. 2004 & 2005	Conversation analysis	31	Paediatric	Not stated	UK	31 Children (6–12 yrs) Parents 16 GPs	High credibility. Limited transferability to similar populations is likely.
Butler 2009 [60]		How nurses deliver advice on telehealth line	Purposive selection of calls. Audio recorded. 2005 –2006.	Conversation analysis	6	Paediatric	Mix	Australia	6 Children 6 Parents 12 Nurses	High credibility. Likely to be transferable to other similar interactions.
Ijas-Kallio 2011 [61]	[62-65]	Patients participation in diagnosis and treatment decision	Audio or video recorded consultations. 2005–2006.	Conversation analysis	46	Mix	RTI	Finland	46 Children Parents 11 Clinicians from 9 clinics	High credibility. Insufficient information to assess transferability or typicality.

### Studies characteristics & relatedness

The 13 included studies reported on a total of 1032 consultations and varied in terms of research question, population, and analysis methods. A detailed description of study characteristics are given in Table 1. Six studies focussed on communication and decision making in consultations for children with RTIs [16,33,35,41,51,61]; three of these studies were very closely related, the later studies building on the earlier studies [16,35,61] while the other three focussed on slightly different aspects of the consultation [33,41,51]. Six studies used conversation analysis (CA) or discourse analysis (DA) to examine the dyadic or triadic communication within the consultation irrespective of the illness (but included at least some acute minor illness) and formed a slightly separate and loosely related group [11,42,45,48,52,53]. The remaining study was rather different to the others in that it looked at communication on the telephone between parents and primary care nurses [60].

### Study quality

None of the studies were high quality with respect to all criteria. Quality assessment was constrained to some extent by all studies reporting insufficient detail, but was a particular issue for two studies for which only brief reports were available [41,51]. The studies which used CA (or a CA informed approach) were generally deemed to have high credibility (internal validity). However, transferability was often limited since the practical constraints of collecting recordings of consultations often meant that a convenience or opportunistic approach had been taken to sampling. The strongest studies with respect to transferability are those by Stivers [16,35] which report phenomena which occur at high frequencies across large samples and which found similar phenomena in two separate studies with demographically differing populations. Four studies [33,41,51,52] were designed to explore particular phenomena and not intended to be transferable, as their authors acknowledge. For a further four studies, it was impossible to assess transferability due to incomplete reporting of sampling method and/or population characteristics [11,48,53,61]. A summary description of the quality of each study in relation to the Cochrane Qualitative Research Methods Group [26] criteria is given in Table 1.

### Translation of second order constructs

We identified 29 second order constructs across the 13 studies which fell into 5 thematically related groups of constructs. Three groups of constructs were associated with a particular stage of the consultation similar to those defined in the model by Charles et al. [66]. This model of the consultation was found to provide a useful framework for organising these 3 major themes: communication during information exchange; communication during diagnosis delivery; and communication about treatment deliberation and decision. The other two groups of constructs related to the role of the parent(s) and of the child in consultation. Table 2 gives an example of how the summary translations were produced from the second order constructs. Table 3 gives a summary translation or description of each construct or theme within each of the five thematically related groups of constructs.

**Table 2:** Example of how summary translation is produced from second order constructs

**Study**	**Second order construct**	**Summary translation**
**Elwyn 1999 **[33]	The doctor has attempted to use the concept of ‘normality’ as a means of persuading the patients to accept symptomatic treatment. It is to be expected that young children will develop upper RTI, and the doctor wants to avoid its medicalization.	Clinicians use problem minimising/normalising language or communication techniques during examination to communicate that an illness is not serious
**Stivers 2000 **[35]	When doctors initiate closure of a minimal sequence (either by moving to a new sequence or with a minimal sequence expanding SCT) they convey that the response is routine, expectable, or unproblematic.	
**Rollnick 2001 **[41]	The doctor in the example above (involving the ‘very rattle cough’) used minimizing words, not only to reassure a worried parent and to reduce the intrusiveness of the physical examination, but also to introduce the idea that the problem was not *that* serious.	
**Butler 2009 **[60]	The nurse draws on her expertise in the area of child development and parenting to re-specify the problem as non-medical and as an expected and normal occurrence.	
	The nurse assures the caller that 37 is ‘normal’ and at ‘37.4 she’s probably feeling a little bit uncomfortable but that’s okay’.	

**Table 3:** Second order constructs from included studies organised into major thematic groups

**Thematically related groups**	**Translation or summary descriptions of second order constructs from included studies**	**Studies which identify 2nd order construct**
**Communication during information exchange**	Parents displayed concern with establishing the ‘doctorability’ of the child’s illness by presenting a story of extreme or abnormal events, and seeking clinicians expertise.	Elwyn 1999 [33]
		Stivers 2000 [35]
		Rollnick 2001 [41]
	Four types of problem presentations have been identified and include ‘symptoms only’, ‘candidate diagnosis’, ‘diagnosis implicative symptoms descriptions’ and ‘candidate diagnosis as background information’.	Stivers 2000 [35]
		Ijas-Kallio 2011 [61]
	Clinicians use problem minimising/normalising language or communication techniques during examination to communicate that an illness is not serious.	Elwyn 1999 [33]
		Stivers 2000 [35]
		Rollnick 2001 [41]
		Butler 2009 [60]
	Clinicians justified ‘no antibiotic’ treatment decisions using problem minimising language as a pre-emptive move to signal a pending ‘no antibiotic’ treatment decision.	Rollnick 2001 [41]
	Parents and clinicians usually communicate purely in the voice of ‘strictly medicine’ (i.e. as though the problem was purely medical) in consultations for simple acute problems (communication phenomenon appears to be specific to these types of cases rather than clinician specific). Communicating only in the voice of medicine contributes to a failure of communication when parents have concerns that cannot be accommodated by this voice.	Barry 2001 [45]
	Clinicians’ communication may be based on an assumption of a patient-centred approach to decision making but parents who do not expect a patient centred approach may misunderstand it and in turn the confusion may contribute to a clinician assessment of a parent as anxious	Roberts 2005 [52]
	Clinician communication about prognosis varied, if duration was mentioned it was often too short or unclear, parents were invited to re-consult ‘if not happy’.	Butler 2004 [51]
**Communication during diagnosis delivery**	Clinicians responded to symptoms only problem presentations of simple acute illness with straightforward unilateral diagnosis announcements presented as being based on his/her own medical reasoning.	Stivers 2000 [35]
		Ijas-Kallio 2011 [61]
	The parent’s problem presentation affects the trajectory of the interaction. When parents gave or implied a candidate diagnosis, the doctor designed his/her reply to be responsive to the parents’ own problem presentation, either confirming or disconfirming the candidate diagnosis.	Stivers 2000 [35]
		Ijas-Kallio 2011 [61]
	Parents and clinicians alike oriented to diagnoses as within the clinician’s domain of expertise. Parents might respond minimally to simple unilateral diagnosis pronouncements but by doing so treat the unilateral decision as adequate.	Stivers 2000 [35]
		Ijas-Kallio 2011 [61]
	Parents might also claim access to diagnostic reasoning by extended responses which might 1) assess the decision positively, 2) evaluate the grounds on which the doctor’s decision is acceptable, or 3) resist the decision.	Ijas-Kallio 2011 [61]
**Communication during treatment deliberation & decision**	Parents usually accepted treatment recommendations.	Stivers 2000 [35]
		Ijas-Kallio 2011 [61]
	Parents resisted by withholding acceptance of treatment recommendations. Parents also drew on their own knowledge of symptoms, past experiences, previous medical advice and diagnostic expectations to contest clinicians’ interpretations.	Stivers 2000 [35]
		Main 2001^1^[42]
		Stivers 2005 [35]
		Ijas-Kallio 2011 [61]
	Overt requests or parent pressure for antibiotics were rare but included: parents making requests for or stating clear preference for antibiotic treatment and parents ’threatening’ to re-consult if antibiotics were not prescribed. More common were enquiries about antibiotics or mentions of positive past experience with antibiotic treatment.	Elwyn 1999 [33]
		Stivers 2000 [35]
		Main 2001^1^[42]
	Clinicians sometimes presented the treatment decision (no antibiotics, delayed prescription, immediate prescription) as a choice to parents; clinician actively pursued parental acceptance of decisions; parents behaved as though they have the right to accept or reject treatment proposals.	Elwyn 1999 [33]
		Stivers 2000 [35]
		Rollnick 2001 [41]
	When parents gave or implied a candidate diagnosis as part of their problem presentation, these were responded to by clinicians in a way which indicated clinicians perceived an expectation for antibiotic treatment from parents, and their responses often included justifications of non-antibiotic treatment.	Stivers 2000 [35]
		Main 2001 [42]
	Clinicians responded to parent resistance in a way which indicated clinicians perceived this as an indication of an expectation for antibiotic treatment from parents.	Stivers 2000 [35]
		Main 2001^1^[42]
	Parent's usually avoided open disagreement; rather they offered alternative or additional info and sought to further the shared understanding of the child’s condition.	Ijas-Kallio 2011 [61]
	Clinicians used various strategies to pursue parental agreement with non-antibiotic treatment including offering symptom relief, further testing, offering parent choice and invoking parental competence	Stivers 2000 [35]
		Rollnick 2001 [41]
		Stivers 2005 [35]
	When clinicians made affirmative, specific, non-minimised treatment recommendations e.g. for symptom relief, parents were less likely to resist and clinicians were more likely to gain acceptance than if clinicians made recommendations against a treatment.	Rollnick 2001 [41]
		Stivers 2005 [35]
	Clinicians acknowledge uncertainty in diagnosis and treatment decision and prescribed antibiotics	Elwyn 1999 [33]
		Rollnick 2001 [41]
	Clinicians met parents preference for antibiotic treatment or responded to parent pressure for antibiotics despite appearing to diagnose a viral condition.	Elwyn 1999 [33]
		Stivers 2000 [35]
**Role of parent in consultation**	Parents gave and received information about their child’s health, illness and context, with parent’s involvement progressively decreasing through adolescence.	Main 2001 [42]
	Parents often asserted themselves during the consultation and until they had been able to express their concerns, would interrupt child-doctor interaction	Main 2001 [42]
		Cahill 2007 [11]
**Role of child in consultation**	Children were notably quiet in these consultations	Cahill 2007 [11]
	Adults determined the degree of the child’s integration in the consultation interaction by the varying degree to which they oriented to or ignored the child. Clinicians affected child participation by varying how they arranged the room or how much they addressed the child rather than the parent or used appropriate communication techniques (asking closed questions, by giving children enough time to respond). Sometimes, both adults co-constructed a situation where the child was treated as a non-person (where child’s contributions were ignored or negated by adults). There was also an intermediate integration where child contributions were acknowledged but not integrated into the discussion.	Tates 2005
		Nova 2005 [53]
		Cahill 2007 [11]
	Where child was integrated he/she made relevant contributions and could influence diagnostic course	Nova 2005 [53]
	Child actively acquired knowledge of the illness and the consultation process during consultations	Nova 2005 [53]

^1^reported in Scott 2001.

### Synthesis of constructs into key themes

1. *Communication during information exchange*

Different styles of problem presentation were identified by Stivers [35] and Ijas–Kallio [61]; the most common was ‘symptoms only’, where the parent described the symptoms that prompted the consultation. The other styles indicated that the parent had a ‘candidate diagnosis’ in mind. Stivers [35] interpreted the ‘candidate diagnosis’ as conveying a stance that the diagnosis is known and therefore the reason for the consultation is to seek treatment, whereas Ijas–Kallio [61] interpreted it as the parent adopting an active epistemological position in the consultation by laying claim to possessing medical knowledge.

During the information exchange, parents and clinicians were often talking at cross purposes about the ‘seriousness’ of the child’s illness. Parents sought to justify the need for the consultation and the need for a clinical evaluation by presenting accounts of extreme or abnormal events [35,41]. However, when clinicians judged an illness was minor, they used problem minimising or normalising language to communicate this during the history taking and physical examination [33,35,41,60]. Rollnick et al. [41] additionally observed that clinicians did so as a pre–emptive move in anticipation of a no antibiotic treatment decision. Clinicians may hear parents’ efforts to establish the need for consultation as indicating an expectation of antibiotics and parents may hear clinicians’ minimising and normalising statements as questioning the need for a consultation, thus both parties may feel challenged and respond accordingly.

Underlying assumptions about the form of the communication [45] or interaction [52] in the consultation contributed to miscommunication where these were not shared by the parents and clinicians. Clinicians usually spoke only in the ‘voice of medicine’ in these consultations [45]. The ‘voice of medicine’ implies an underlying assumption of a biomedical model of the problem and is contrasted with the ‘voice of the life–world’, which encompasses the patient’s life, world and normal social interaction [67]. Where parents had concerns that were not easily expressed in terms of a simple medical problem, these could also lead to miscommunication. Some ‘lifeworld’ concerns expressed by parents in response to diagnosis/treatment decision delivery were interpreted as resistance and as an indication of a desire for antibiotics by clinicians e.g. concern about missing school [35] or desire to be well for an impending holiday [43]. An underlying assumption of a biomedical model of the problem constrained the communication between clinicians and parents in these consultations.

2. *Communication during diagnosis delivery*

Parents and clinicians almost always oriented to diagnosis as within the clinician’s domain of expertise. The most common form of diagnosis communication, regardless of the diagnostic label, was a straightforward exchange: pronouncement by the clinician and acceptance, with minimal response, by the parent [35,61]. Stivers [35] and Ijas-Kallio [61] observed that parents’ problem presentations (‘symptoms only’ or ‘candidate diagnosis’) affected the trajectory of the interaction. ‘Symptoms only’ elicited a straightforward diagnosis delivery but when a candidate diagnosis is offered or implied, the clinician always responded to either confirm or disconfirm. Ijas-Kallio [61] further observed that parents sometimes lay claim to diagnostic reasoning through an extended response to the diagnosis which gives the parents’ views on the acceptability of this diagnosis and sometimes supports and sometimes resists the diagnosis.

3. *Communication during treatment deliberation and decision*

There were many cases where the decision making followed a simple pattern in which the clinician gave a unilateral diagnosis/treatment decision based on their own medical reasoning, which the parent accepted [35,61]. Such consultations are described by Rollnick et al. [41] as having a sense of “business as usual”. This straight forward form of unilateral decision making occurred in both cases of antibiotic treatment and no antibiotic treatment. Parents often responded minimally but in a way which treated the unilateral pronouncements as adequate [35,61] or occasionally (as for the diagnosis) parents responded more extensively to assess the decision, by drawing on their own knowledge or previous experience to support or challenge the decision [61]. Thus unilateral decision making could be a co-constructed activity which did not restrict the parents’ ability to participate in the decision making process [61]. This implies that in many of these cases, parents expect a unilateral decision by the clinician rather than a shared decision making process.

Three studies observed that both clinicians and parents oriented towards the treatment decision as negotiable [33,35,41]. Clinicians used various strategies to pursue parental acceptance of treatment recommendations [16,35,41] and were most successful with positive, specific, non-minimised recommendations [16,41]. Where clinicians and parents differed as to whether or not antibiotic treatment was needed, it was not always possible to achieve either an accepted unilateral decision or a negotiated shared decision and the desires of one party had to give way to the other [33]. In the reported data are examples of both parents giving way to clinicians and *visa versa*. Where clinicians gave an antibiotic prescription despite an initial viral diagnosis, they justified it either on the grounds of uncertainty in relation to diagnosis [33,41] or in response to perceived parent pressure [33,35].

The form of parents’ problem presentation could influence communication of the treatment recommendations. Stivers [35] reports that clinicians perceive candidate diagnosis presentations as pressure or expectation of antibiotic treatment and their recommendations therefore included justifications of non-antibiotic treatment. Scott et al. [43] go further and interpret candidate diagnosis as a way in which parents express pressure for antibiotics, although Stivers et al. [14] state that it is not possible to infer intention. In addition, a range of parent communications and behaviours during diagnosis and treatment communication were interpreted by clinicians as resistance [16,35,43,61] and responded to by clinicians as pressure for antibiotics [35,43]. However, much of what is interpreted as parental resistance consists of parents’ presenting or requesting additional information relating to particular symptoms, diagnostic expectations and/or past experiences and in most cases the parents had not indicated a pre-consultation expectation of antibiotics [14]. Ijas-Kallio [61] observes that parental resistance usually sought to further a shared understanding of the child’s condition. It seems possible that when parents seek additional information at this late stage in the consultation, clinicians may misinterpret this as pressure for antibiotics. Barry et al. [45], the only included study to have conducted post consultation interviews with parents, identified two cases where misinterpretation of parental information requests had resulted in prescription of unwanted and unnecessary antibiotics.

4. *Role of parent in consultation*

Parents were the main providers and receivers of information about children’s health problems [42] in the data from all the included studies. Parental involvement was progressively less with older (adolescent) children but parents were very dominant in consultations with children up to 12 years. Parents often asserted themselves during the consultation [42] and clearly needed to express their concerns for their child’s health, often interrupting any clinician-child interaction until they had been able to do so [11].

5. *Role of child in consultation*

Children were described as noticeably quiet in these consultations [11] and children’s voices were rare in the transcripts reproduced in the included studies. The adults in the interaction (parent and clinician) together determined the extent to which the child could participate by varying the degree to which they orient to or ignore the child [11,48,53]. Although minimal, children’s contributions were meaningful and useful, often including some account of their illness experience or asking relevant questions [53]. There was evidence of children learning about the illness and about how consultations work [53].

## Discussion

This is the first review to synthesise the qualitative evidence on the interaction in primary care consultations for children with acute minor illness. It provides insights into the usual forms of communication between parents, children and clinicians in primary care consultations and in particular how this influences the antibiotic decision making process in consultations for acute RTI. The focus in the literature on consultations for RTI, stimulated by concerns about over prescription of antibiotics, meant there was insufficient evidence to comment on the decision making in relation to other acute illnesses.

A key finding of this review is that parent concern or information seeking may be misinterpreted by clinicians as pressure for antibiotics. Pressure or expectation for antibiotics has been cited by clinicians and researchers as playing a major role in over-prescription of antibiotics [6,43,68,69]. However, clinician perception of parental expectation of antibiotics is not associated with actual parent expectations [36]. This review found that explicit parental pressure was rare but many parental communication behaviours were interpreted by clinicians as pressure for antibiotics. These behaviours were, at least, ambiguous in this respect. The interpretation by clinicians of a wide range of parental communication behaviours as expectation or pressure for antibiotics, and clinicians’ pre-emptive moves to justify no antibiotic prescription, indicated that many clinicians anticipated pressure for antibiotics. This may explain why clinicians read an expectation of antibiotics into parental communication that is only intended to communicate concerns or to elicit further information. Since clinician perception of an expectation of antibiotics is a strong predictor of whether antibiotics are prescribed [36] it is essential to understand the process by which these perceptions are created. This review provides evidence that parents’ concerns about their child and clinicians’ anticipation of antibiotic expectation can combine to contribute to over-prescription of antibiotics.

A common misunderstanding observed in these consultations occurred when parents and clinicians spoke about the ‘seriousness’ of the illness. They often spoke at cross purposes, the parent seeking to justify the consultation (my child is seriously ill) and the clinician to justify a no antibiotic treatment decision (this illness will resolve without intervention). Problem minimising commentaries (“online commentaries”) during physical exams are frequently used by clinicians in consultations for acute illness, mainly to reassure patients [37]. In addition, online commentaries which identify problems or abnormalities rather than minimise or normalise symptoms are associated with higher rates of antibiotic prescribing [56]. Clinicians contribute to the miscommunication by predominantly using problem minimising communication in association with viral diagnoses so that when parents perceive any signs or symptoms that appear non-minor, they may then have a greater expectation of antibiotics [54]. Problem minimising communication by the clinician may have a different influence on the consultation trajectory depending on whether it is perceived as reassurance that a child’s illness is minor or as questioning the legitimacy of the consultation or as indicating a disregard of the parent’s concerns. In the latter cases, it may prompt parent resistance which in turn can influence antibiotic prescribing.

The child’s contribution to communication, when present, was meaningful and indicated that the child was also seeking to understand their illness and the consultation process.

In common with the findings of previous reviews [23,70], this review found that data on children’s voices in consultations were rare. This may be partly explained by the age profile of children most frequently consulting for cough which peaks at under 18 months [1] when most children have limited communication capacity. However, this review did include consultations with children up to 12 years old and the data indicate that when these children do speak, the extent of their contribution is controlled by the adults [11,48,53]. An important question for future research concerns the children’s views of the communication in these consultations. In particular whether children are learning that they do not have a say and what that means for their understanding of self-care in relation to cough.

### Limitations

Few of these studies captured the views and intentions of parents or clinicians. This meant that we can comment on the talk itself, but not the intent and thus ambiguities in communication remain. Studies which capture both the interaction in the consultation and the views of all parties are needed.

None of these studies gave detailed socio-demographic information about the entire population (clinician, parent and child), many reporting on only clinician or parent populations and sometimes neither. It was therefore not possible to know whether particular phenomena are associated with certain socio-demographic groups. Further we cannot comment on the extent to which the patterns of illness or prescribing in the observed consultations were typical or atypical, but instead comment on features of the observed exchanges.

## Conclusions

Key areas for improving communication in consultations for children with RTIs are identified by this review. Communication skills training has been shown to reduce antibiotic prescribing significantly for RTI in adults [17-19] and a booklet used to aid communication was shown to reduce antibiotic prescribing for RTIs in children [71]. This review provides strong evidence that most parental communication should be heard as expressions of concern, contributions of information or attempts at understanding and not as requests for antibiotics. These consultations for common acute illness may appear straightforward biomedical problems to clinicians, but by taking a more bio-psycho-social approach clinicians may avoid some of the miscommunication. In interventions with adult patients which successfully reduced prescribing, a key element of the communication skills training focuses on improving clinician elicitation of concerns [17-19]. This review suggests that this approach could be usefully transferred to consultations for children, with additional training of clinicians and to interpret parent communication as expressions of concern or attempts at understanding. In addition, problem minimising or normalising language should be used with caution to avoid unhelpful miscommunication about the ‘seriousness’ of the child’s illness. Clinicians and parents need to communicate more explicitly about expectations for antibiotics in order to avoid unnecessary and unsought prescriptions and to address any misapprehensions about when antibiotics are needed. The key communication skill is the clinician’s ability to elicit the real expectations of parents when they consult with a sick child.

### Consent

Written informed consent was obtained from the patient’s guardian/parent/next of kin by the authors of the included studies for the publication in those reports.

## Competing interests

The authors declare that they have no competing interests.

## Authors’ contributions

CC, JH, AH and PL were responsible for developing the research questions and study design; PL,CC, JH for study management and analysis; CC, JH, AH and PL writing the manuscript; CC accepted the final version. All authors read and approved the final manuscript.

## Pre-publication history

The pre-publication history for this paper can be accessed here:

http://www.biomedcentral.com/1471-2296/15/63/prepub

## Supplementary Material

Additional file 1 Supplementary Material: Medline Search Strategy. Click here for file
