# Supplementary material for: How communication affects prescription decisions in consultations for acute illness in children: a systematic review and meta-ethnography
Source: BMC Fam Pract. 2014 Apr 8;15:63. doi: 10.1186/1471-2296-15-63 (PMC4234398; doi:10.1186/1471-2296-15-63)
Supplement: Additional file 1 — Supplementary Material: Medline Search Strategy. [file 1471-2296-15-63-S1.doc]

**Additional file**

**Supplementary Material: Medline Search Strategy**

1 exp Respiratory Tract Infections/ (253292)

2 (sore throat or tonsillitis or pharyngitis or nasopharyngitis or laryngitis).af. (17842)

3 ("respiratory tract infection" or "respiratory tract infections").af. (33394)

4 (rti$ or urti$ or lrti$ or "chest infection" or "chest infections").af. (18646)

5 exp Cough/ (10284)

6 exp Respiration Disorders/ (127246)

7 (bronchitis or bronchiolitis or pneumonia or croup).af. (119633)

8 (tracheitis or sinusitis or rhino-sinusitis).af. (18119)

9 (cough or "common cold" or influenza or flu).af. (94695)

10 exp Fever/ (30280)

11 (fever or febrile or "high temperature").af. (148950)

12 ("acutely ill" or "acute illness" or "acute illnesses").af. (5127)

13 ("respiratory infection" or "respiratory infections").af. (12456)

14 "ill health".af. (2776)

15 (acute adj3 illness).af. (5460)

16 Otitis Externa/ or Otitis Media with Effusion/ or Otitis/ or Otitis Media, Suppurative/ or Otitis Media/ (21568)

17 (otitis or ear ache or earache).af. (25847)

18 exp Earache/ (495)

19 10 or 11 (148969)

20 ((Fever or (fever or febrile or "high temperature")) not malaria).af. (144763)

21 ((Fever or (fever or febrile or "high temperature")) not malaria not typhoid).af. (135727)

22 1 or 2 or 3 or 4 or 5 or 6 or 7 or 8 or 9 or 12 or 13 or 14 or 15 or 16 or 17 or 18 or 21 (617936)

23 "breathing difficulty".af. (146)

24 (conjunctivitis or "eye infection" or "eye infections").af. (18925)

25 (vomiting or diarrh$).af. (116584)

26 ("urinary tract infection" or uti).af. (14348)

27 22 or 23 or 24 or 25 or 26 (747103)

28 ("ear infection" or "ear infections").af. (1050)

29 "glue ear".af. (263)

30 ("ear disease" or "ear diseases").af. (8471)

31 (otalgia or ottorhoea or ottorhea or "hearing loss").af. (36077)

32 (rhinosinusitis or rhinitis or tonsillopharyngitis).af. (28226)

33 (difficulty adj3 breathing).af. (611)

34 (dyspnea or dysponea).af. (26510)

35 congestion.af. (8930)

36 (consolidation adj3 lung).af. (290)

37 (minor adj3 illness).af. (342)

38 (self-limiting or "self limiting").af. (4154)

39 27 or 28 or 29 or 30 or 31 or 32 or 33 or 34 or 35 or 36 or 37 or 38 (817382)

40 limit 39 to (humans and ("infant (1 to 23 months)" or "preschool child (2 to 5 years)" or "child (6 to 12 years)")) (160763)

41 exp Parents/ (57676)

42 exp parent-child relations/ or exp father-child relations/ or exp mother-child relations/ or exp parenting/ (43677)

43 exp Child/ (1364726)

44 (child$ or school child$ or schoolchild$ or preschool$ or pre-school$ or infant$ or infancy or todder$ or baby or babies).af. (2106768)

45 (parent$ or mother$ or father$ or mum$ or mom$ or dad$ or carer$ or caregiver$).af. (570262)

46 age.ab. or age.ti. (1116442)

47 limit 46 to ("infant (1 to 23 months)" or "preschool child (2 to 5 years)" or "child (6 to 12 years)") (271843)

48 Pediatrics/ or Infant/ or Child, Preschool/ (909717)

49 (paediatric$ or pediatric$).af. (590042)

50 41 or 42 or 43 or 44 or 45 or 47 or 48 or 49 (2550232)

51 exp "Patient Acceptance of Health Care"/ (130168)

52 exp Maternal Behavior/ (8509)

53 50 or 51 or 52 (2659489)

54 exp Primary Health Care/ (62156)

55 exp Family Practice/ (59615)

56 exp Physicians, Family/ (14272)

57 (doctor$ or Dr$ or GP$ or clinician$ or ((health adj2 professional$) or practitioner$)).af. (3230081)

58 ("primary care" or "primary health care").af. (91392)

59 (walk-in centre or walkin centre or walk-in centres or walkin centres).af. (89)

60 ((general adj1 practice$) or (general adj1 practioner$) or (family adj1 practice$) or (family adj1 practioner$)).ab. (20939)

61 ((general adj1 practice$) or (general adj1 practioner$) or (family adj1 practice$) or (family adj1 practioner$)).ti. (19960)

62 60 or 61 (34523)

63 54 or 55 or 56 or 57 or 58 or 59 or 62 (3333817)

64 exp Ambulatory Care/ (41248)

65 63 or 64 (3362422)

66 exp "Referral and Consultation"/ (49130)

67 exp Office Visits/ (4597)

68 exp House Calls/ (1990)

69 (consult$ or reconsult$ or visit$ or revisit$ or re-consult$or re-visit$).af. (232689)

70 exp Communication/ (309750)

71 (help adj3 seek$).af. (4163)

72 (care adj3 seek$).af. (5686)

73 exp Physician-Patient Relations/ (52478)

74 (communicat$ or discuss$ or advice or advise)af. (1172500)

75 66 or 67 or 68 or 69 or 70 or 71 or 72 or 73 or 74 (1613975)

76 (repeat$ adj3 consultat$).af. (80)

77 75 or 76 (1613975)

78 conservation$.af. (63861)

79 77 or 78 (1670970)

80 (health adj3 seek$).af. (3186)

81 attend$.af. (86303)

82 79 or 80 or 81 (1733862)

83 exp Qualitative Research/ (9640)

84 exp Interview, Psychological/ or exp Interview/ (31146)

85 exp Focus Groups/ (10984)

86 (interview$ or questionnair$ or survey$ or "focus group" or "focus groups").af. (703733)

87 (narration or narrative$).af. (10853)

88 (attitude$ or view$ or belief$ or viewpoint$ or perception$ or standpoint$).af. (709917)

89 (Nudist or Nvivo or Atlas ti).af. (428)

90 ("behavioural research" or "behavioral research").af. (19992)

91 ("qualitative research" or "qualitative study" or "qualitative method" or "qualitative methodology" or "qualitative design" or "qualitative methods").af. (21111)

92 ("grounded theory" or "action research" or "content analysis" or "thematic analysis").af. (13252)

93 psycholog$.af. (627977)

94 ethnopsycholog$.af. (475)

95 ethnolog$.af. (1766)

96 survey$.af. (335971)

97 ("audiotape recording" or "tape recording" or taperecording* or "tape recorded").af. (5092)

98 "qualitative data".af. (3560)

99 anthropolog$.af. (29079)

100 ("audio recording" or "audio recordings" or "digital audio").af. (343)

101 83 or 84 or 85 or 86 or 87 or 88 or 89 or 90 or 91 or 92 or 93 or 94 or 95 or 96 or 97 or 98 or 99 or 100 (1704947)

102 40 and 53 and 65 and 82 and 101 (1693)
